# *AINTEGUMENTA-LIKE* genes have partly overlapping functions with *AINTEGUMENTA* but make distinct contributions to *Arabidopsis thaliana* flower development

**DOI:** 10.1093/jxb/erv224

**Published:** 2015-05-08

**Authors:** Beth A. Krizek

**Affiliations:** Department of Biological Sciences, University of South Carolina, Columbia, SC 29208, USA

**Keywords:** *AINTEGUMENTA-LIKE*, *Arabidopsis thaliana*, carpel patterning, flower development, organ growth, organ initiation, petal, sepal fusion, unequal genetic redundancy.

## Abstract

*AINTEGUMENTA* (*ANT*) is an important regulator of *Arabidopsis* flower development that has overlapping functions with the related *AINTEGUMENTA-LIKE6* (*AIL6*) gene in floral organ initiation, identity specification, growth, and patterning. Two other *AINTEGUMENTA-LIKE* (*AIL*) genes, *AIL5* and *AIL7*, are expressed in developing flowers in spatial domains that partly overlap with those of *ANT*. Here, it is shown that *AIL5* and *AIL7* also act in a partially redundant manner with *ANT*. The results demonstrate that *AIL* genes exhibit unequal genetic redundancy with roles for *AIL5*, *AIL6*, and *AIL7* only revealed in the absence of *ANT* function. *ant ail5* and *ant ail7* double mutant flowers show alterations in floral organ positioning and growth, sepal fusion, and reductions in petal number. In *ant ail5*, petals are often replaced by filaments or dramatically reduced in size. *ant ail7* double mutants produce increased numbers of carpels, which have defects in valve fusion and a loss of apical tissues. The distinct phenotypes of *ant ail5*, *ant ail7* and the previously characterized *ant ail6* indicate that *AIL5*, *AIL6*, and *AIL7* make unique contributions to flower development. These distinct roles are also supported by genetic analyses of *ant ail* triple mutants. While *ant ail5 ail6* triple mutants closely resemble *ant ail6* double mutants, *ant ail5 ail7* triple mutants exhibit more severe deviations from the wild type than either *ant ail5* or *ant ail7* double mutants. Furthermore, it is shown that *AIL5*, *AIL6*, and *AIL7* act in a dose dependent manners in *ant* and other mutant backgrounds.

## Highlight

Two additional members of the *Arabidopsis AINTEGUMENTA-LIKE* (*AIL*) transcription factor gene family, *AIL5* and *AIL7*, are shown to have overlapping functions with *ANT* in floral organ development.

## Introduction

Flowers arise on the flanks of the inflorescence meristem at the sites of auxin maxima (Reinhardt *et al.*, 2003). Within these flower primordia, floral organ primordia are initiated at characteristic positions within concentric rings called whorls. The sites of floral organ initiation within a flower appear to correspond to auxin maxima, although it is not clear if auxin specifies the founder cell population or accumulates after these cells are specified and promotes organ outgrowth (Chandler *et al.*, 2011). After initiation, these primordia adopt a sepal, petal, stamen or carpel fate as a consequence of the activities of distinct combinations of four classes of floral organ identity genes (reviewed in Krizek and Fletcher, 2005). In whorl one, class A and E genes specify sepal identity. In whorl two, class A, B, and E genes specify petal identity. In whorl three, class B, C, and E genes specify stamen identity, while in whorl four, class C and E genes specify carpel identity. Most of the class A, B, C, and E genes encode MADS domain transcription factors that form tetrameric complexes regulating distinct target genes in each whorl of the flower (Smaczniak *et al.*, 2012). Furthermore, these MADS domain protein complexes appear to act throughout floral organ development activating both early and later targets during floral organogenesis (Ito *et al.*, 2004, 2007; Kaufmann *et al.*, 2010; Wuest *et al.*, 2012; O’Maoleidigh *et al.*, 2013).

**Fig. 1.**
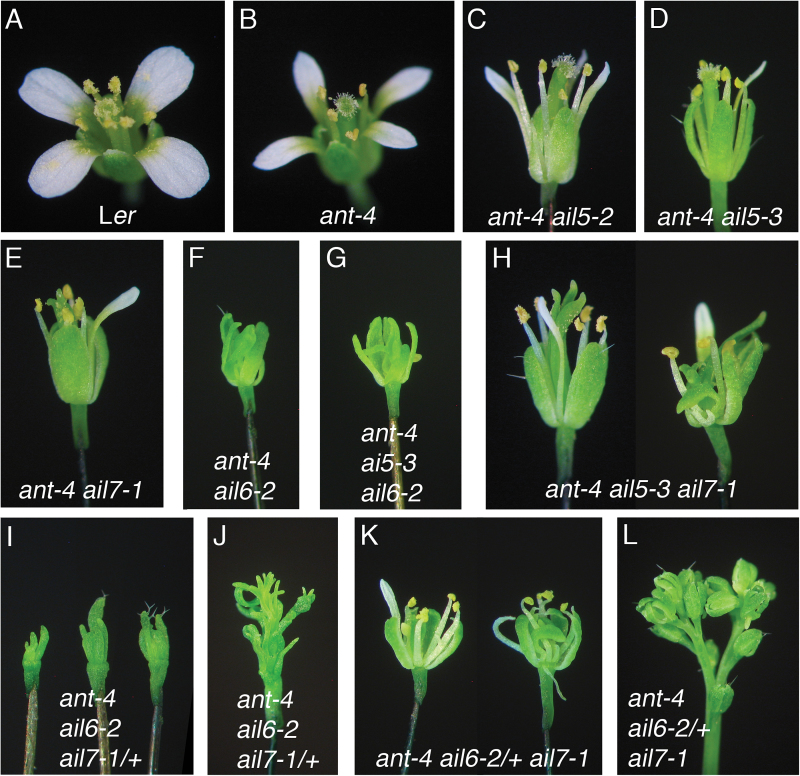
*AIL5* and *AIL7* have partially overlapping functions with *ANT* in flower development. (A) L*er* flower. (B) *ant-4* flower. (C) *ant-4 ail5-2* flower. (D) *ant-4 ail5-3* flower. (E) *ant-4 ail7-1* flower. (F) *ant-4 ail6-2* flower. (G) *ant-4 ail5-3 ail6-2* flower. (H) Early-arising (left) and later-arising (right) *ant-4 ail5-3 ail7-1* flowers. (I) *ant-4 ail6-2 ail7-1/+* flowers. (J) *ant-4 ail6-2 ail7-1/+* inflorescence. (K) Early-arising (left) and later-arising (right) *ant-4 ail6-2/+ ail7-1* flowers. (L) *ant-4 ail6-2/+ ail7-1* inflorescence.

Members of the AINTEGUMENTA-LIKE/PLETHORA (AIL/PLT) family of transcription factors play important roles in many developmental processes in plants including flower development (reviewed in Horstman *et al.*, 2014). AILs are a small subgroup of eight proteins within the large AP2/ERF transcription factor family (see Supplementary Fig. S1 at *JXB* online). The founding member of the family, AINTEGUMENTA (ANT), is a key regulator of floral organ growth. Mutations in *AINTEGUMENTA* (*ANT*) result in flowers with smaller floral organs while ectopic expression of *ANT* results in floral organs that reach a larger final size (Elliott *et al.*, 1996; Klucher *et al.*, 1996; Krizek, 1999; Mizukami and Fischer, 2000). In addition, *ant* mutants have ovule defects and are female sterile (Elliott *et al.*, 1996; Klucher *et al.*, 1996). Three other *AIL* genes: *AIL5*, *AIL6*, and *AIL7* are expressed in developing flowers at lower levels than *ANT* but in spatial domains that partially overlap that of *ANT* (Nole-Wilson *et al.*, 2005) (see Supplementary Fig. S1 at *JXB* online). Loss of *AIL5*, *AIL6* or *AIL7* function by itself has no phenotypic consequences on flower development (Nole-Wilson *et al.*, 2005; Krizek, 2009; Prasad *et al.*, 2011). In the case of *AIL6*, this is due to genetic redundancy with *ANT* (Krizek, 2009). *ant ail6* flowers exhibit severe defects in floral organ positioning, identity specification, growth, and patterning. These flowers consist primarily of small sepals, filaments, and unfused carpel valves; they lack petals and stamens and a normal gynoecium (Fig. 1F). The floral organ identity defects in *ant ail6* flowers are likely to be a consequence of reduced expression of class B and C genes during the early stages of flower development.

While roles for *AIL5* and *AIL7* in floral organ development have not been described, these two genes have been shown to regulate shoot phyllotaxy and lateral root initiation in combination with *AIL6*. In *plt3 plt5 plt7* (i.e. *ail5 ail6 ail7*) triple mutants, flower initiation deviates from spiral phyllotaxy with two adjacent flowers often separated by 90° or 180° rather than 137.5° (Prasad *et al.*, 2011). The positioning of lateral roots is also disrupted in this triple mutant with clusters of lateral roots arising in the triple mutant rather than being distributed along the root longitudinal axis (Hofhuis *et al.*, 2013). In addition to these roles in primordium positioning, *AIL5* also regulates germination and seedling growth (Yamagishi *et al.*, 2009; Yano *et al.*, 2009). Furthermore, ectopic expression of *AIL5* results in the formation of embryo-like structures on seedlings (Tsuwamoto *et al.*, 2010). This phenotype is similar to that resulting from over-expression of *BABY BOOM* (*BBM*), another member of the *AIL* family (Boutilier *et al.*, 2002). *AIL5* misexpression also results in the production of larger flowers, similar to the phenotype of plants over-expressing *ANT* (Nole-Wilson *et al.*, 2005).

To investigate the possible roles of *AIL5* and *AIL7* in flower development further, double and triple mutants were made. *ant ail5* and *ant ail7* double mutants show more severe phenotypes than *ant* single mutants indicating that *AIL5* and *AIL7* have partially redundant functions with *ANT* during flower development. While *ant ail5*, *ant ail6* and *ant ail7* double mutants share some phenotypic similarities, each double mutant has a unique appearance. This suggests that *AIL5*, *AIL6*, and *AIL7* make distinct contributions to flower development in the absence of *ANT*. This conclusion is further supported by the unique phenotypes of *ant ail* triple mutants. Furthermore, it was found that *AIL5, AIL6*, and *AIL7* act in a dose-dependent manner in the *ant* background. Dose dependent behaviour is also observed for *AIL6* and *AIL7* in the *ant ail7* and *ant ail6* backgrounds, respectively. Floral organ development is, for the most part, normal in *ail5 ail6 ail7* plants, although the petals are slightly smaller in size. These results indicate that these three genes only contribute to flower development in the absence of *ANT*. These results demonstrate unequal genetic redundancy among members of the *AIL* gene family.

## Materials and methods

### Plant materials and growth conditions

Mutants used in the study were *ant-4* (Baker *et al.*, 1997; Nole-Wilson and Krizek, 2006), *ail5-2* (Prasad *et al.*, 2011), *ail5-3* (identified in this study), *ail6-2* (Krizek, 2009), and *ail7-1* (Krizek, 2009). Plants were grown on a soil mixture of Metro-Mix 360:perlite:vermiculite (5:1:1 by vol.) in 16h days (100–150 μmol m^–2^ s^–1^) at a temperature of 20–22 °C.

### Genetics and PCR genotyping

*ant-4* is in the Landsberg *erecta* (*er*) background while the *ail5-2*, *ail5-3*, *ail6-2*, and *ail7-1* alleles were originally in the Columbia background. Double and triple mutants were identified in the F_2_ or later generations by PCR genotyping. All double and triple mutants characterized were those carrying the *er* allele. *ant-4* was PCR genotyped as described previously (Krizek, 2009). Genotyping of *ail5-2*, *ail5-3*, *ail6-2*, and *ail7-1* was performed using the primers listed in Supplementary Table S1 at *JXB* online. The *ail5-2 ail6-2 ail7-1* and *ail5-3 ail6-2 ail7-1* triple mutants phenotypically characterized were those containing *er* from crosses of double mutants carrying *er*.

### SEM

Tissue for SEM was fixed, dried, dissected, and coated as described previously (Krizek, 1999). SEM analyses were performed on a Tescan Vega 3 SBU variable pressure SEM.

### Petal measurements

Petal measurements were performed on at least 12 petals from flower at positions 1–10 on an inflorescence from at least four different plants. Petal measurements were performed essentially as described previously (Trost *et al.*, 2014). Petals from approximately stage 13 flowers (at the time when the long stamens were at the same height as the carpel) were removed with forceps and placed on Sellotape. Once all petals were collected, the tape was stuck to a piece of black plexiglass and scanned at a resolution of 3600 dpi in 8-bit greyscale. Petal area, length, and width were determined using Image J software.

### Gynoecium clearing

Gynoecium clearing was performed as described previously (Wynn *et al.*, 2014). Briefly, the tissue was fixed in ethanol:acetic acid (9:1 v/v) for 2h at room temperature and rinsed twice in 90% ethanol. The tissue was transferred to Hoyer’s solution (70% chloral hydrate, 5% gum arabic, 4% glycerol) for several hours, dissected, and mounted.

### RNA extraction, RT-PCR and real time PCR

RNA was extracted from inflorescences using Trizol following the manufacturer’s instructions with cleanup on an RNeasy column (Qiagen). The RNA was DNased while on the column. First strand cDNA synthesis was performed using Quanta qScript cDNA SuperMix (Quanta BioSciences) following the manufacturer’s instructions. RT-PCR and real-time PCR was performed using the primers listed in Supplementary Table S1 at *JXB* online. The RT-PCR experiment usd the following PCR conditions: 40 cycles of 92 °C for 30 s, 55 °C for 30 s, and 72 °C for 2min followed by 1 cycle of 72 °C for 5min. Real-time PCR was performed on a BioRad iCycler as described previously (Krizek and Eaddy, 2012).

## Results

### Mutations in *AIL5* have no effect on flower development but enhance *ant* single mutants

Several *ail5* alleles have been described previously. *ail5-1* corresponds to a transgenic inverted repeat *AIL5* knockdown line (Nole-Wilson *et al.*, 2005). *ail5*-2 (i.e. *plt5-2*) contains a T-DNA insertion within the eighth exon of the gene (Prasad *et al.*, 2011) (see Supplementary Fig. S2A at *JXB* online). In addition, three *ail5* alleles (*cho1-1*, *cho1-2*, and *cho1-3*) have been described (Nambara *et al.*, 2002; Yamagishi *et al.*, 2009). Another allele (*ail5-3*) that contains a T-DNA insertion within the first intron of the gene was identified (see Supplementary Fig. S2A at *JXB* online) (Alonso *et al.*, 2003). Like the previously identified *ail5-1* and *ail5-2* alleles, *ail5-3* produces normal flowers (see Supplementary Fig. S2B–D at *JXB* online). No full-length transcript was produced in *ail5-3*, although partial transcripts corresponding to sequences downstream of the T-DNA insertion site were detected (see Supplementary Fig. S2E, F at *JXB* online). Transcripts corresponding to sequences upstream of *ail5-2* were also detected, indicating that neither *ail5-2* nor *ail5-3* are RNA null alleles (see Supplementary Fig. S2F at *JXB* online).

**Fig. 2.**
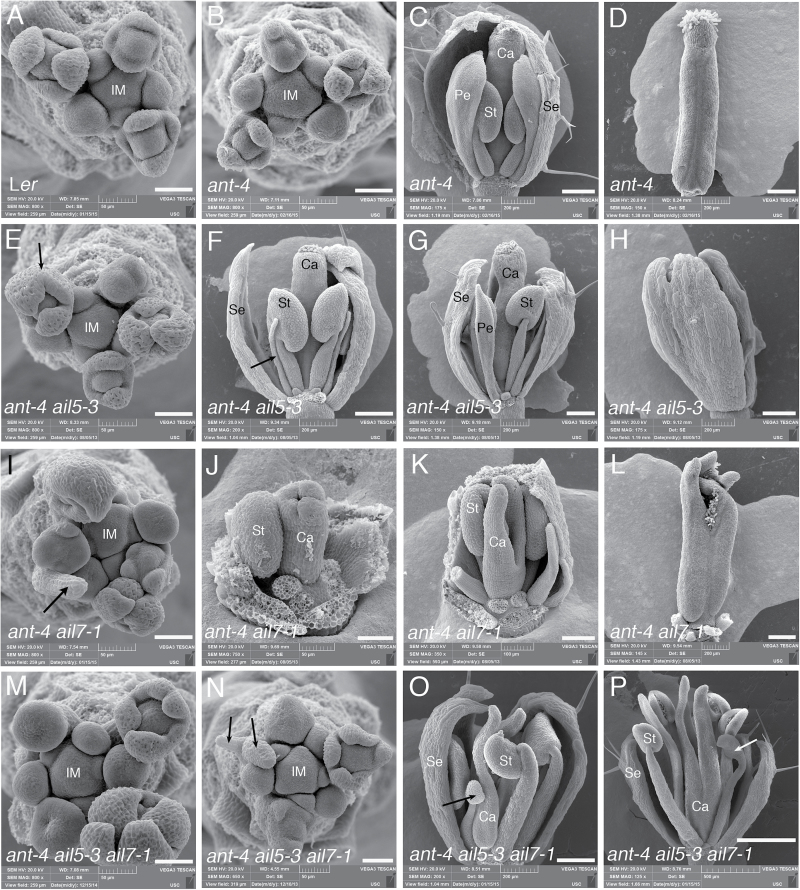
Scanning electron micrographs of L*er*, *ant-4* and *ant-4 ail* mutant combinations. (A) L*er* inflorescence meristem. (B) *ant-4* inflorescence meristem. (C) *ant-4* flower. (D) *ant-4* carpels. (E) *ant-4 ail5-3* inflorescence meristem. Arrow points to the reduced boundary between two adjacent sepal primordia. (F) *ant-4 ail5-3* flower with filaments in the second whorl in place of petals. Arrow points to one filament. (G) *ant-4 ail5-3* flower with a thin petal in the second whorl. (H) *ant-4 ail5-3* flower with fused sepals. (I) *ant-4 ail7-1* inflorescence meristem. Arrow points to filament-like structure that arises in place of a flower. (J) Young *ant-4 ail7-1* flower showing abnormal development of the fourth whorl carpels. (K) *ant-4 ail7-1* flower with two unfused carpels in the fourth whorl. (L) *ant-4 ail7-1* carpels in which the valves are unfused at their apex and some stigmatic tissue arises at their edges. (M, N) *ant-4 ail5-3 ail7-1* inflorescence meristems. Arrows in (N) point to filament-like structures that arise in place of flowers. (O, P) *ant-4 ail5-3 ail7-1* flowers with stamenoid organs (arrows). IM, inflorescence meristem; Se, sepal; Pe, petal; St, stamen; Ca, carpel. Scale bars, 50 μm (A, B, E, I, J, M, N), 100 μm (K) 200 μm (C, D, F–H, L, O), 500 μm (P).

To investigate whether *AIL5* acts redundantly with *ANT*, *ant-4 ail5-2* and *ant-4 ail5-3* double mutants were constructed. Both double mutants show a more severe second whorl phenotype compared with *ant-4*. *ant-4 ail5-2* flowers produce similar numbers of petals as *ant-4* but these petals are smaller in size (Fig. 1A–C; Tables 1, 2). *ant-4 ail5-3* flowers produce many fewer petals than *ant-4*; most second whorl organs are missing or replaced with filaments (Figs 1D, 2C, F; Table 1). The petals that are present are very thin (Fig. 2G; Table 2). The *ant-4 ail5-3* double mutant was characterized in more detail.

**Table 1.** *Floral organ counts in* Ler, ant, ant ail5, ant ail7, *and* ant ail5 ail7 *flowers at positions 1–30 on the inflorescence*

	L*er* 1–30	*ant-4* 1–30	*ant-4 ail5-2* 1–30	*ant-4 ail5-3* 1–30	*ant-4 ail7-1* 1–30	*ant-4 ail5-3 ail7-1* 1–30
**Whorl 1**						
Se	4.0	3.99	4.02	3.78	4.01	4.41
Filament				0.01		
Curled white				0.03		0.01
**Total**	**4.0**	**3.99**	**4.02**	**3.82**	**4.01**	**4.41**
**Whorl 2**						
Pe	4.0	3.74	3.91	0.56	1.72	0.93
Filament		0.02		0.91	0.05	0.49
St/Pe		0.01	0.01			0.05
**Total**	**4.0**	**3.77**	**3.92**	**1.47**	**1.77**	**1.47**
**Whorl 3**						
St	5.77	4.38	4.87	4.24	4.82	4.56
St-like						0.24
Filament			0.01	0.02		0.28
Pe/St			0.01		0.06	0.18
**Total**	**5.77**	**4.38**	**4.89**	**4.26**	**4.88**	**5.26**
**Whorl 4**						
Ca	2.0	2.0	2.0	2.0	2.35	2.32
**Total**	**2.0**	**2.0**	**2.0**	**2.0**	**2.35**	**2.32**
**Total of all whorls**	**15.77**	**14.14**	**14.83**	**11.55**	**13.01**	**13.46**

**Table 2.** Petal area, width, and length in various genotypes Genotypes grouped together were grown and measured at the same time.

	Petal area (mm^2^)	Petal length (mm)	Petal width (mm)
L*er*	1.82±0.18	2.95±0.19	0.97±0.09
*ant-4*	0.94±0.11	2.47±0.18	0.59±0.07
*ant-4 ail5-2*	0.48±0.07	2.14±0.13	0.28±0.04
*ant-4 ail5-3*	0.40±0.10	2.24±0.22	0.20±0.05
*ant-4 ail7-1*	1.02±0.21	2.40±0.21	0.66±0.09
*ant-4 ail5-3 ail7-1*	0.59±0.09	2.33±0.18	0.32±0.05
*ant-4*	0.83±0.09	2.38±0.14	0.58±0.05
*ant-4 ail5-3/+*	0.68±0.09	2.28±0.18	0.49±0.06
L*er*	1.77±0.27	2.85±0.23	1.03±0.09
*ail5-2 ail6-2 ail7-1*	1.38±0.19	2.31±0.11	0.94±0.09
*ail5-3 ail6-2 ail7-1*	1.31±0.18	2.27±0.17	0.93±0.06

**Fig. 3.**
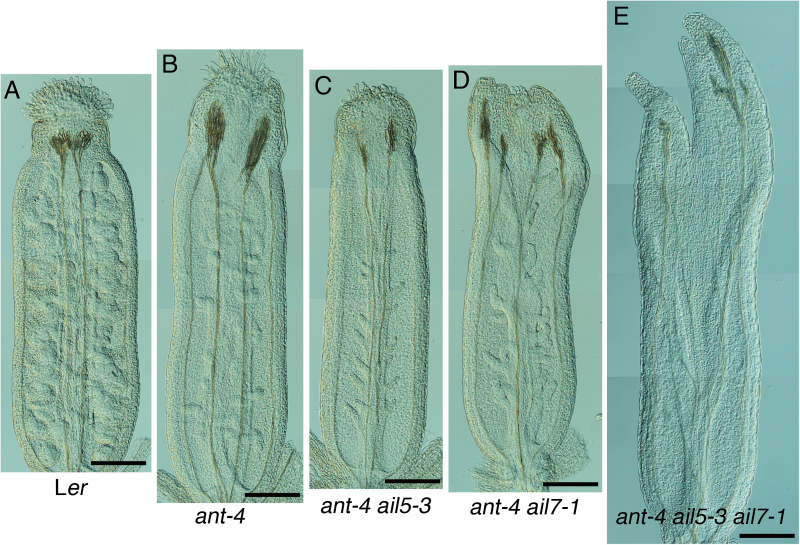
*AIL5* and *AIL7* contribute to gynoecium development. (A) L*er* gynoecium. (B) *ant-4* gynoecium. (C) *ant-4 ail5-3* gynoecium. (D) *ant-4 ail7-1* gynoecium. (E) *ant-4 ail5-3 ail7-1* gynoecium. Scale bars, 200 μm.

**Fig. 4.**
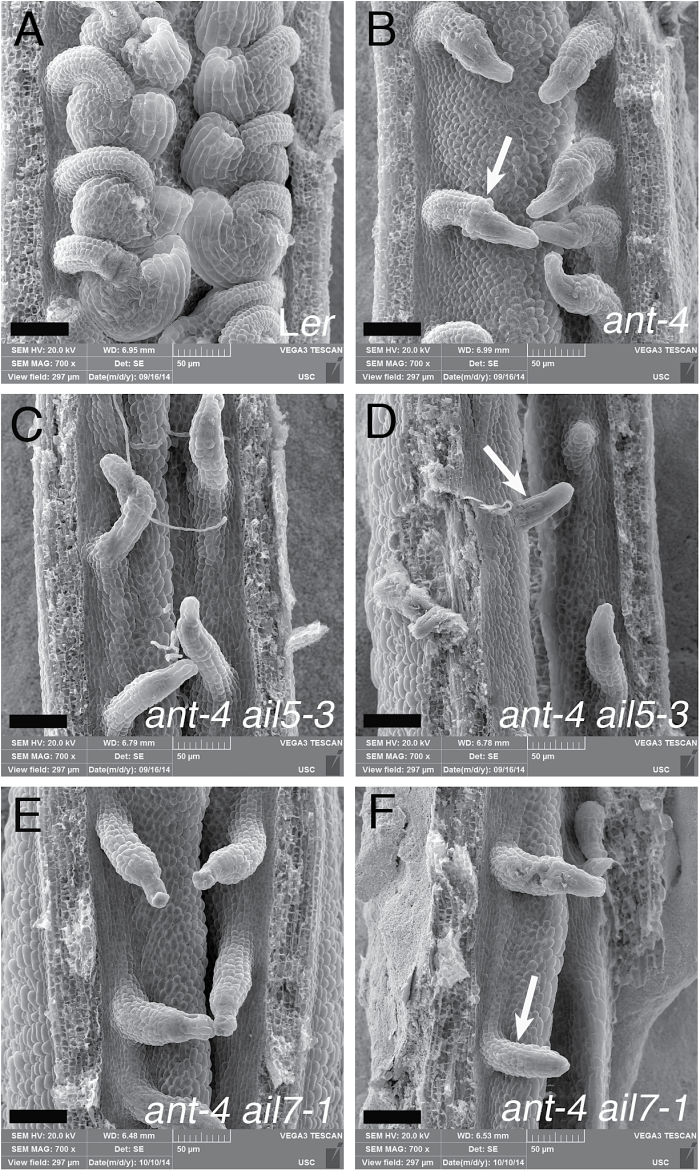
*AIL5* and *AIL7* contribute to ovule development. (A) L*er* ovules. (B) *ant-4* ovules. Arrow points to swelling in chalazal region. (C, D) *ant-4 ail5-3* ovules. Arrow in (D) points to small ovule primordium that lacks regional distinctions. (E, F) *ant-4 ail7-1* ovules. Arrow in (F) points to small ovule primordium that lacks regional distinctions. Scale bars, 50 μm.

In addition to petal defects, *ant-4 ail5-3* flowers exhibit more severe defects in whorls one and four compared with *ant-4*. *ant-4 ail5-3* flowers show an increase frequency of sepal fusion (Table 3) and scanning electron microscopy of *ant-4 ail5-3* flowers reveals altered patterns of sepal initiation and growth (Fig. 2A, B, E). Sepal primordia do not always arise in the characteristic adaxial and abaxial positions separated by 180 °C (Fig. 2E). Boundaries between adjacent sepals are not established in some flowers leading to the growth of two adjacent sepals as a fused structure (Fig. 2E, H). Examination of a cleared *ant-4 ail5-3* gynoecium suggested that the double mutant might exhibit a more severe defect in ovule development than *ant-4* (Fig. 3A–C). While *ant-4* ovules do not initiate integuments (Baker *et al.*, 1997), they often show a slight swelling in the chalazal region (Fig. 4A, B); this swelling was not observed in *ant-4 ail5*-3 ovules (Fig. 4C, D). In addition, some *ant-4 ail5-3* ovule primordia were very short with no cellular distinction of regional identities (nucellus, chalaza, funiculus) along the apical-basal axis of the ovule (Fig. 4D).

**Table 3.** *Floral organ fusion in* Ler, ant, ant ail5, ant ail7, *and* ant ail5 ail7 *flowers at positions 1–30 on the inflorescence*

	L*er* 1–30	*ant-4* 1–30	*ant-4 ail5-3* 1–30	*ant-4 ail7-1* 1–30	*ant-4 ail5-3 ail7-1* 1–30
% of flowers with Se fusion	0	2.0	46.3	43.6	39.4
% of flowers with St fusion	0	10.1	8.05	11.4	7.34
% of flowers with unfused Ca	0	3.0	2.0	96.7	95.5
% of flowers with Ca more than halfway unfused	0	0	0	37.3	66.6
% of flowers with Ca lacking stigmatic tissue	0	2.2	0	69.3	74.8

### Mutations in *AIL7* enhance *ant* single mutants

Previously, mutations in *AIL7* were shown to have no phenotypic consequence on flower development (Krizek, 2009). In addition, it was shown that *AIL7* did not act redundantly with its closest homologue *AIL6*. To investigate possible genetic redundancy between *ANT* and *AIL7*, the *ant-4 ail7-1* double mutant was made. *ant-4 ail7-1* flowers show an enhanced phenotype in whorls one, two, and four compared with *ant-4* (Fig. 1B, E). Like *ant-4 ail5-3*, the *ant-4 ail7-1* double mutant displays an increased frequency of sepal fusion in the first whorl (Table 3) and altered initiation and growth of sepal primordia (Fig. 2I). In the second whorl, *ant-4 ail7-1* double mutants produce fewer petals than *ant-4* (Table 1). These petals are similar in area to *ant-4* petals (Table 2). In the fourth whorl, *ant-4 ail7-1* carpels are unfused to varying extents (Table 3; Fig. 2D, K, L). In approximately one-third of flowers, the fusion defects extend more than halfway down the length of the carpel values (Table 3; Fig. 2K). Stigmatic tissue is often reduced or absent in *ant-4 ail7-1* carpels (Figs 2L, 3D; Table 3). Defects in carpel growth are evident early in development of the fourth whorl (Fig. 2J). *ant-4 ail7-1* mutants also appear to have slightly more severe ovule defects than *ant-4*. *ant-4 ail7-1* ovule primordia exhibit less pronounced swelling of the chalazal region and are sometimes quite short and lack regional distinctions, similar to *ant-4 ail5-3* ovules (Fig. 4E, F). In addition to the floral organ defects mentioned above, flower initiation from the inflorescence meristem was occasionally disrupted with some flowers being replaced by filament-like structures in which floral organs were not initiated (Fig. 2I).

**Fig. 5.**
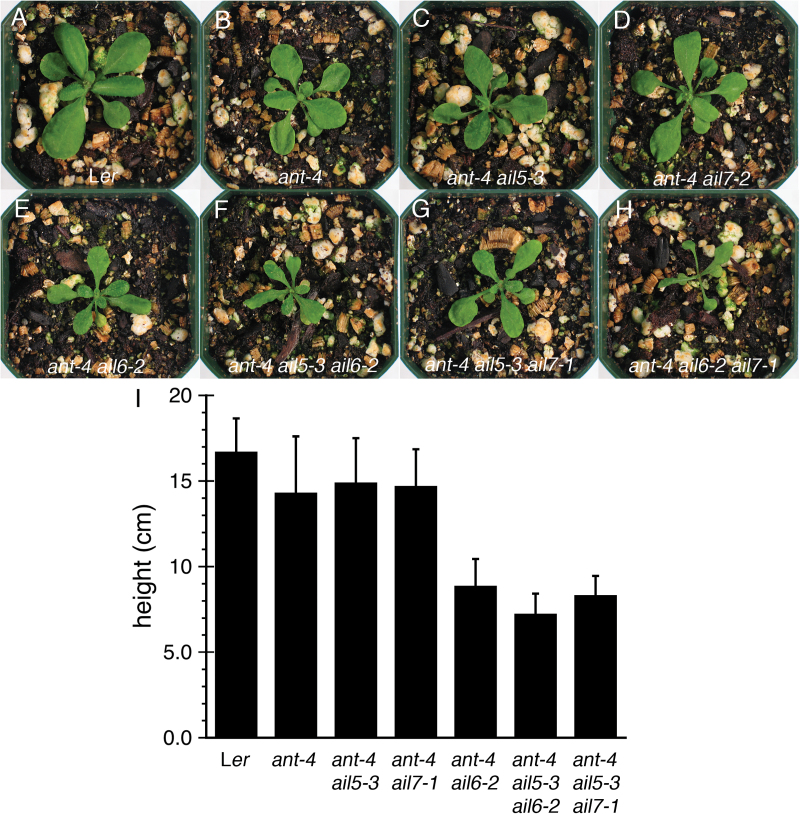
*AIL5* and *AIL7* contribute to leaf development and shoot growth. (A). L*er* plant. (B) *ant-4* plant. (C) *ant-4 ail5-3* plant. (D) *ant-4 ail7-1* plant. (E) *ant-4 ail6-2* plant. (F) *ant-4 ail5-3 ail6-2* plant. (G) *ant-4 ail5-3 ail7-1* plant. (H) *ant-4 ail6-2 ail7-1* plant. All plants in (A–H) were 20-d-old. (I) Graph of plant heights. All plants were 42-d-old.

The vegetative phenotypes of *ant-4 ail5-3* and *ant-4 ail7-1* double mutants were also investigated. No dramatic differences in rosette leaf size or plant height were observed in either of these double mutants compared with *ant-4* (Fig. 5A–D, I). This is in contrast to *ant-4 ail6-2* double mutants that produce smaller rosette leaves and shorter plants compared with *ant-4* (Fig. 5E, I).

### *ant ail5 ail6* triple mutant flowers resemble *ant ail6* double mutant flowers

To investigate the functions of *AIL5* and *AIL7* further, *ant-4 ail5-3 ail6-2* and *ant-4 ail5-3 ail7-1* triple mutants were made. *ant-4 ail6-2 ail7-1* triple mutants were shown previously not to make flowers due to the termination of the shoot apical meristem during vegetative development (Fig. 5H) (Mudunkothge and Krizek, 2012). *ant-4 ail5-3 ail6-2* triple mutant flowers resemble *ant-4 ail6-2* flowers with regard to floral organ types with no petals or true stamens present in either genotype (Fig. 1F, G; Table 4). Instead both triple mutants produce flowers that primarily consist of sepals, stamenoid organs, and unfused carpel valves (Table 4). *ant-4 ail5-3 ail6-2* triple mutants do show enhanced leaf and shoot phenotypes compared with *ant-4 ail6-2*. The triple mutant produces smaller leaves and the plants are shorter in height (Fig. 5E, F, I). This indicates that while *AIL5* does not make additional contributions to flower development in the absence of *ANT* and *AIL6*, it does contribute to leaf and shoot growth in the absence of these genes.

**Table 4.** *Floral organ counts in* ant-4 ail6-2 *and* ant-4 ail5-3 ail6-2 *flowers at positions 1–20 on the inflorescence*

	*ant-4 ail6-2* 1–20	*ant-4 ail5-3 ail6-2* 1–20
Se	4.24	3.92
Flat white	0.02	
Filament	0.78	1.56
Swollen filament	0.60	0.37
Flat green	0.02	0.06
Stamenoid organs	1.30	2.15
St/Va	0.35	0.17
Ca Valve like (unfused)	2.04	2.56
**Total**	**9.35**	**10.79**

### *ant ail5 ail7* triple mutants show more severe vegetative and reproductive phenotypes than either double mutant

Overall, *ant-4 ail5-3 ail7-1* triple mutant flowers show more severe deviations from the wild type than either *ant-4 ail5-3* or *ant-4 ail7-1* double mutants (Fig. 1D, E, H). The triple mutant flower phenotype is complex with aspects that are novel, additive, epistatic or synergistic compared with either double mutant. *ant-4 ail5-3 ail7-1* flowers contain more sepals than either double mutant (Table 1) but with similar amounts of sepal fusion as the two double mutants (Table 3). Similar to both *ant-4 ail5-3* and *ant-4 ail7-1* double mutants, the pattern and growth of sepal primordia is also altered in *ant-4 ail5-3 ail7-1* triple mutants (Fig. 2M, N). In the second whorl of the triple mutant, the numbers of petals and filaments are intermediate between the two double mutants (Table 1). Petal size in *ant-4 ail5-3 ail7-1* is also intermediate between *ant-4 ail5-3* and *ant-4 ail7-1* (Table 2). In the third whorl, there is more variation in organ type than observed in either double mutant with some filaments, stamen-like and petaloid stamens present in addition to normal stamens (Table 1; Fig. 2O, P). With regard to carpel number in the fourth whorl, the triple mutant resembles *ant-4 ail7-1* (Table 1); however, there is a more dramatic loss of carpel valve fusion in the triple mutant (Table 3). Approximately two-thirds of *ant-4 ail5-3 ail7-1* flowers contain carpels that are unfused for more than half of their length; this is almost twice the number observed in *ant-4 ail7-1*. These gynoecia only rarely produce ovule primordia (Fig. 3E).

In addition, the overall morphology of the floral organs and organization of the triple mutant flowers degrade with developmental time such that later-arising flowers appear increasingly disorganized (Fig. 1H). Stamens in later-arising flowers often do not make pollen, exhibit altered morphology (Fig. 2O, P), and are reduced in size. As described for *ant-4 ail7-1* inflorescences, *ant-4 ail5-3 ail7-1* inflorescences also show the replacement of some flowers with thick filamentous structures (Fig. 2N). *ant-4 ail5-3 ail7-1* plants possess narrower leaves and are reduced in height compared with *ant-4 ail5-3* and *ant-4 ail7-1* double mutants (Fig. 5C, D, G, I).

### Dose-dependent behaviour of *AIL6* and *AIL7* in the *ant ail7* and *ant ail6* backgrounds

**Fig. 6.**
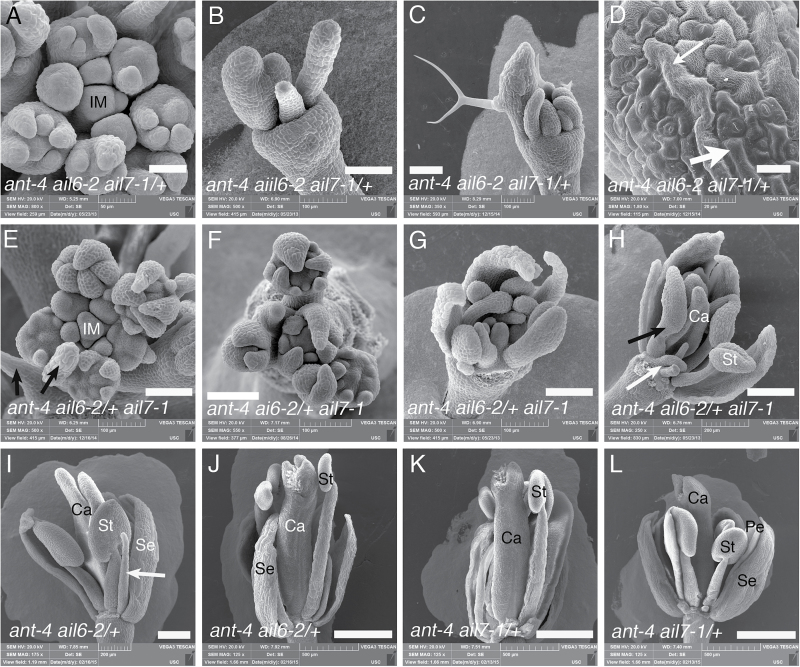
*AIL6* and *AIL7* act in a dose-dependent manner as shown by scanning electron micrographs. (A) *ant-4 ail6-2 ail7-1/+* inflorescence meristem. (B, C) *ant-4 ail6-2 ail7-1/+* flowers. (D) Surface of a flat organ arising in the periphery of an *ant-4 ail6-2 ail7-1/+* flower. Both giant cells characteristic of sepals (thin white arrow) and leaf-like cells (thick white arrow) are present. (E) *ant-4 ail6-2/+ ail7-1* inflorescence meristem. Arrows point to filaments that arise in place of flowers. (F) Inflorescence apex of *ant-4 ail6-2/+ ail7-1* plant. No inflorescence meristem is visible. (G) *ant-4 ail6-2/+ ail7-1* flower with many organ primordia that arise in altered positions and lack normal morphologies. (H) *ant-4 ail6-2/+ ail7-1* flower with stamenoid organ (black arrow). Short filaments are present in this flower (white arrow). (I) *ant-4 ail6-2/+* flower with unfused carpel valves and a second whorl filament (arrow). (J) *ant-4 ail6-2/+* flower with carpels that are unfused at their apex. (K, L) *ant-4 ail7-1/+* flowers showing loss of carpel fusion at the apex of the gynoecium. IM, inflorescence meristem; Se, sepal; Pe, petal; St, stamen; Ca, carpel. Scale bars, 20 μm (D), 50 μm (A), 100 μm (B, C, E–G), 200 μm (H, I), 500 μm in (J–L).

Because *ant-4 ail6-2 ail7-1* plants do not possess shoot apical meristems that persist long enough to produce flowers, the effect of the combined loss of *ANT*, *AIL6*, and *AIL7* on flower development cannot be examined. It was decided to investigate the consequences of the loss of a single copy of *AIL6* or *AIL7* in a background compromised for *ANT* and either *AIL7* or *AIL6* activity, respectively. *ant-4 ail6-2 ail7-1/+* plants produce flowers that are more severe than *ant-4 ail6-2* with fewer and smaller organs (Figs 1I, 6 A–C). *ant-4 ail6-2 ail7-1/+* flowers produced 5.5±1.5 organs per flower while *ant-4 ail6-2* flowers produced 9.5±1.9 organs. Most *ant-4 ail6-2 ail7-1/+* floral organs are green and filamentous with overall morphologies that lack a resemblance to a normal floral organ (Figs 1I, 6B, C). Some of the organs arising in the outer region of these flowers contain giant cells characteristic of sepals as well as leaf-like epidermal cells (Fig. 6D). *ant-4 ail6-2 ail7-1/+* inflorescences terminate with the production of filaments in place of flowers (Fig. 1J).

The phenotypic consequences of the loss of a single copy of *AIL6* in the *ant-4 ail7-1* background were investigated next. *ant-4 ail6-2/+ ail7-1* plants produce flowers that have more severe defects than *ant-4 ail7-1* (Fig. 1E, K). *ant-4 ail6-2/+ ail7-1* flowers produce more sepals but fewer petals, stamens, and carpels, with some of these second, third, and fourth whorl organs replaced by filaments (Table 5). Floral organ initiation patterns and the growth of floral organ primordia are more severely disrupted than in *ant-4 ail7-1* flowers (Fig. 6E–G). Like *ant-4 ail5-3 ail7-1*, the *ant-4 ail6-2/+ ail7-1* floral phenotype becomes more severe with developmental age. Later-arising *ant-4 ail6-2/+ ail7-1* flowers exhibit some loss of normal floral organ morphologies (Figs 1K, 6H). *ant-4 ail6-2/+ ail7-1* plants also show several inflorescence meristem defects. Filaments are sometimes produced in place of flowers (Fig. 6E). In addition, the inflorescence meristem often terminates with the production of flowers (Fig. 6F). In other cases, the inflorescence meristems get wider, splitting in half with both halves of the meristem continuing to initiate new flowers (Fig. 1L).

**Table 5.** *Floral organ counts in* ant-4 ail7-1 *and* ant-4 ail6-2/+ ail7-1 *flowers at positions 1–20 on the inflorescence*

	*ant-4 ail7-1* 1–20	*ant-4 ail6-2/+ ail7-1* 1–20
**Whorl 1**		
Se and Se like	3.97	5.36
**Whorl 2**		
Pe and Pe-like	2.00	0.51
Filament	0.04	0.62
St/Pe	0.04	0.01
**Total**	**2.08**	**1.14**
**Whorl 3**		
St and St-like	5.04	4.35
Filament	0.02	0.94
Pe/St	0.05	0.01
**Total**	**5.11**	**5.30**
**Whorl 4**		
Ca and Va like	2.60	1.10
Filament		0.31
St/Va		0.12
**Total**	**2.60**	**1.53**
**Total of all whorls**	**13.76**	**13.33**

### Dose-dependent behaviour of *AIL5*, *AIL6*, and *AIL7* in the *ant* background

Any possible dosage effects of *AIL5* were also investigated by examining flower development in *ant-4 ail5-3/+* flowers. *ant-4 ail5-3/+* flowers show a reduction in petal numbers with the replacement of a few petals with filaments but these defects are less severe than *ant-4 ail5-3* double mutants (Tables 1, 6). The decrease in petal number gets more severe in later-arising flowers (Table 6). In addition, petals are slightly smaller than *ant-4* but larger than in *ant-4 ail5-3* (Table 2) indicating that the *AIL5* dose does matter in a background lacking *ANT* activity.

**Table 6.** *Floral organ counts in* ant *and* ant ail5/+ *flowers at positions 1–30 on the inflorescence*

	*ant-4* 1–10	*ant-4* 11–20	*ant-4* 21–30	*ant-4 ail5-3/+* 1–10	*ant-4 ail5-3/+* 11–20	*ant-4 ail5-3/+* 21–30
**Whorl 1**						
Se	4.00	3.98	3.98	4.05	4.00	3.95
Filament				0.03		
**Total**	**4.00**	**3.98**	**3.98**	**4.08**	**4.00**	**3.95**
**Whorl 2**						
Pe	4.00	3.73	3.50	3.38	3.13	2.58
Filament		0.05	0.05	0.28	0.28	0.45
**Total**	**4.00**	**3.78**	**3.55**	**3.66**	**3.41**	**3.03**
**Whorl 3**						
St	4.23	4.25	4.30	4.38	4.58	4.38
**Total**	**4.23**	**4.25**	**4.30**	**4.38**	**4.58**	**4.38**
**Whorl 4**						
Ca	2.00	2.00	2.00	2.00	2.00	2.00
**Total**	**2.00**	**2.00**	**2.00**	**2.00**	**2.00**	**2.00**
**Total of all whorls**	**14.23**	**14.01**	**13.83**	**14.12**	**13.99**	**13.36**

It was also found that *AIL6* and *AIL7* exhibit dose-dependent behaviour in the *ant* mutant background. For both *AIL6* and *AIL7*, the respective *ant ail/+* combination shows a phenotype intermediate between that of *ant* and the corresponding *ant ail* double mutant. *ant-4 ail6-2/+* flowers produce fewer petals and filaments in place of some petals (Fig. 6I, J). In addition, these flowers show more severe defects in carpel fusion compared with *ant-4* (Fig. 6I, J). Carpel fusion defects are also more common in *ant-4 ail7-1/+* flowers compared with *ant-4*. The lack of carpel fusion in *ant-4 ail7-1/+* flowers is usually restricted to the apex of the carpels (Fig. 6K, L)

### Floral organ development is largely normal in *ail5 ail6 ail7* triple mutants

**Fig. 7.**
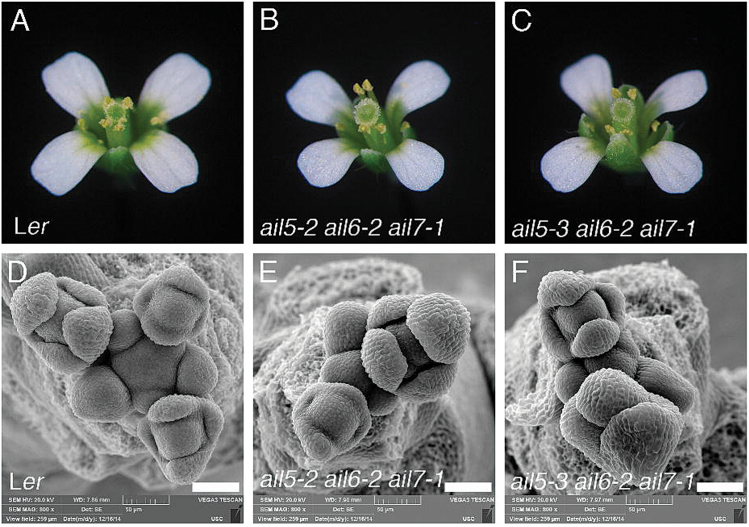
Loss of *AIL5*, *AIL6*, and *AIL7* together has little effect on floral organ development. (A) L*er* flower, (B) *ail5-2 ail6-2 ail7-1 er* flower. (C) *ail5-3 ail6-2 ail7-1 er* flower. (D) Scanning electron micrograph of L*er* inflorescence. (E) Scanning electron micrograph of *ail5-2 ail6-2 ail7-1 er* inflorescence. (F) Scanning electron micrograph of *ail5-3 ail6-2 ail7-1 er* inflorescence. Scale bars, 50 μm.

*ail5-2 ail6-1 ail7-1* (i.e. *plt3-1 plt5-2 plt7-1*) triple mutants have previously been reported to exhibit defects in phyllotaxy, with two successive flowers often arising with divergence angles of 90 °C or 180 °C rather than 137.5 °C (Prasad *et al.*, 2011). However, floral organ development has not been described in this triple mutant. Flower development was examined in the *ail5-2 ail6-2 ail7-1* and *ail5-3 ail6-2 ail7-1* triple mutants in the *er* background. These triple mutants did not exhibit dramatic differences in floral organ development compared with wild-type flowers (Fig. 7A–F), although the petals were slightly smaller in the triple mutant compared with L*er* (Table 2). As described previously for *ail5-2 ail6-1 ail7-1* ER, *ail5-2 ail6-2 ail7-1 er* and *ail5-3 ail6-2 ail7-1 er* triple mutants display alterations in the positioning of flower initiation within the inflorescence meristem (Prasad *et al.*, 2011). The inflorescence meristems are surrounded by fewer flower primordia, which initiate at angles nearing 180 °C (Fig. 7D–F).

## Discussion

### *AIL5*, *AIL6* and *AIL7* make distinct contributions to vegetative and reproductive development

Flowers produced by *ant ail5*, *ant ail6*, and *ant ail7* double mutants share some phenotypic similarities. Flowers from all three double mutants show either a reduction in the number of petals (*ant ail5*, *ant ail7*) or a complete absence of petals (*ant ail6*). Defects in petal initiation are observed even with the loss of a single copy of *AIL5* or *AIL6* in the *ant* mutant background. Thus petal initiation seems to be particularly sensitive to AIL activity. In addition, all three *ant ail* double mutants show defects in sepal positioning within the periphery of the flower primordium (Fig. 2; Krizek, 2009). In the case of *ant ail5-3* and *ant-4 ail7-1*, this often leads to the fusion of adjacent sepals. Despite these similarities, the *ant ail5*, *ant ail6*, and *ant ail7* double mutants also exhibit distinct phenotypes, particularly in the inner whorls. Stamen initiation and identity specification as well as carpel positioning, growth, and patterning is only dramatically altered in *ant ail6* (Krizek, 2009). Besides *ant ail6* flowers, defects in carpel fusion also occur in most *ant ail7* flowers, but only rarely in *ant* and *ant ail5* flowers.

These results indicate that *AIL5*, *AIL6*, and *AIL7* make distinct contributions to flower development. Each of these contributions appears to overlap completely with *ANT* function in floral organ development, as no defects are observed in *ail5*, *ail6* or *ail7* single mutants. In the absence of *ANT*, *AIL5* contributes primarily to sepal positioning and growth and petal initiation and growth. By contrast, *AIL6* contributes to primordium positioning and growth throughout the flower, petal and stamen identity specification, and carpel growth and patterning (Krizek, 2009). *AIL7* primarily contributes to sepal positioning, petal initiation, and carpel growth. Thus, *AIL6* makes more important contributions to floral organ development than *AIL5* or *AIL7*. The distinct roles of *AIL5*, *AIL6*, and *AIL7* in flower development are not unexpected given the distinct expression pattern of these genes within developing flowers (see Supplementary Fig. S1 at *JXB* online; Nole-Wilson *et al.*, 2005). However, the particular phenotypes are not necessarily correlated with the spatial expression patterns of each gene. For example, in wild-type flowers, *AIL7* mRNA is present only in the centre of the floral primordium starting at stage 2 of flower development yet *ant ail7* double mutants show defects in the positioning of sepal primordia in the periphery of stage 3 flowers. This could indicate a non-cell autonomous function of *AIL7*. A recent report suggests that PLT proteins can move between cells in the root (Mähünen *et al.*, 2014). Alternatively, the expression pattern of *AIL7* could be altered in the *ant* mutant background.

Increasing loss of *AIL* function in flowers is generally but not always associated with increasingly more severe deviations from wild-type flower development. An exception is the *ant ail5 ail6* triple mutant, which closely resembles the *ant ail6* double mutant suggesting that the *AIL5* function completely overlaps the combined activities of *ANT* and *AIL6*. However, rather than acting in a partially redundant fashion, there are some suggestions of complex genetic interactions among *AIL* genes in flowers. For example, certain aspects of the *ant ail5 ail7* triple mutant phenotype, such as an increased numbers of sepals and stamens, are novel with respect to either double mutant. Other aspects such as petal number and petal size are intermediate in value between the two doubles, suggesting that mutations in *AIL7* partially rescue the petal defects of *ant ail5* double mutants. It is possible that *AIL5* and *AIL7* have somewhat opposing actions in petal growth and may regulate common target genes in opposite directions. Petal area and petal width may be slightly increased in *ant ail7* double mutants compared with *ant* single mutants (Table 1). Complex and possible antagonistic behaviours for *ANT/AIL6* and *AIL7* within the inflorescence meristem have been noted previously (Mudunkothge and Krizek, 2012). Opposing activities of AIL6 and AIL7 in the flower might contribute to the slight rescue of petal defects in the *ant ail5 ail7* triple mutant if AIL6 activity is increased upon the loss of *AIL7*.

*AIL5*, *AIL6*, and *AIL7* all contribute to vegetative growth in the absence of *ANT*. Generally, increasing loss of *AIL* function results in smaller rosette leaves and shorter plants, although *AIL5*, *AIL6*, and *AIL7* do not make equivalent contributions to vegetative growth either. Once again, *AIL6* appears to play a more important role than *AIL5* or *AIL7*, as *ant ail6* double mutant plants are the shortest of the *ant ail* double mutants.

### Unequal genetic redundancy within the *AIL* family

Genetic redundancy occurs as a consequence of gene duplication. The initial full genetic redundancy of duplicated genes often disappears as one gene acquires mutations resulting in nonfunctionalization, neofunctionalization or subfunctionalization. Partial genetic redundancy results when neither gene is sufficient by itself to provide the ancestral function. In the case of unequal genetic redundancy, loss of one of the two genes results in a phenotype, loss of the other gene has no effect, while loss of both genes results in a more severe phenotype. This is the case with the *AIL* gene family in flowers. Loss of the *ANT* function results in a mutant floral phenotype, no mutant phenotype results from the loss of *AIL5*, *AIL6* or *AIL7*, and an enhanced phenotype is observed in *ant ail5*, *ant ail6*, and *ant ail7* double mutants. Such unequal genetic redundancy implies that the trait controlled by the genes depends in a quantitative manner on the sum of the activities of the two genes (Briggs *et al.*, 2006). Although dispensable on its own, the remaining activity provided by the duplicated gene makes a significant contribution to the overall activity of the gene pair as shown by the severity of the phenotype upon the loss of both genes. Unequal genetic redundancy has been reported a number of times in *Arabidopsis* (Briggs *et al.*, 2006).

The unequal redundancy of *AIL* genes in flower development may result from different expression levels and/or expression patterns of the genes. *ANT* is expressed at much higher levels than *AIL5*, *AIL6* or *AIL7* and in a broader domain within developing flowers (Nole-Wilson *et al.*, 2005). Alternatively, the unequal genetic redundancies could be a consequence of different protein activities. It will be interesting to investigate the molecular basis for the unequal redundancies of *AIL* genes in flowers.

Unequal genetic redundancy is not observed in four *AIL* genes that function in the root: *PLETHORA1* (*PLT1*), *PLT2*, *PLT3/AIL6*, and *BBM/PLT4*. These four genes contribute to root growth and root stem cell maintenance in an overlapping and largely additive manner (Galinha *et al.*, 2007). Single mutations in each of these genes has modest effects on root development while higher order mutants show increasingly severe defects with *plt1 plt2/+ plt3 bbm* plants sometimes lacking root and hypocotyl (Aida *et al.*, 2004; Galinha *et al.*, 2007). The two most highly expressed *PLT* genes, *PLT1* and *PLT2*, do make more significant contributions to root development than *PLT3/AIL6* and *BBM/PLT4* (Galinha *et al.*, 2007). These different contributions result primarily from differences in expression and, to some extent, distinct protein activities (Galinha *et al.*, 2007).

## Supplementary data

Supplementary data can be found at *JXB* online.

Supplementary Fig. S1. AIL family tree and summary of gene expression data.

Supplementary Fig. S2. *ail5* mutants have a wild-type appearance.

Supplementary Table S1. Primers used in this study

Supplementary Data

## Abbreviations:

ANT: AINTEGUMENTA
AIL5: AINTEGUMENTA-LIKE5
AIL6: AINTEGUMENTA-LIKE6
AIL7: AINTEGUMENTA-LIKE7
RT-PCR: reverse transcription PCR.
