# Supplementary material for: AINTEGUMENTA-LIKE genes have partly overlapping functions with AINTEGUMENTA but make distinct contributions to Arabidopsis thaliana flower development
Source: J Exp Bot. 2015 May 8;66(15):4537–49. doi: 10.1093/jxb/erv224 (PMC4507765; doi:10.1093/jxb/erv224)
Supplement: Supplementary Data [file supp_66_15_4537__index.html]

 AINTEGUMENTA-LIKE genes have partly overlapping functions with AINTEGUMENTA but make distinct contributions to Arabidopsis thaliana flower development — AINTEGUMENTA-LIKE genes have partly overlapping functions with AINTEGUMENTA but make distinct contributions to Arabidopsis thaliana flower development — Supplementary Data 

# *AINTEGUMENTA-LIKE* genes have partly overlapping functions with *AINTEGUMENTA* but make distinct contributions to *Arabidopsis thaliana* flower development

## Supplementary Data

Data files

**Files in this Data Supplement:**

- Supplementary Data - Supplementary Data
