# Supplementary material for: AINTEGUMENTA-LIKE genes have partly overlapping functions with AINTEGUMENTA but make distinct contributions to Arabidopsis thaliana flower development
Source: J Exp Bot. 2015 May 8;66(15):4537–49. doi: 10.1093/jxb/erv224 (PMC4507765; doi:10.1093/jxb/erv224)
Supplement: Supplementary Data [file supp_erv224_jexbot147678_file001.pdf]

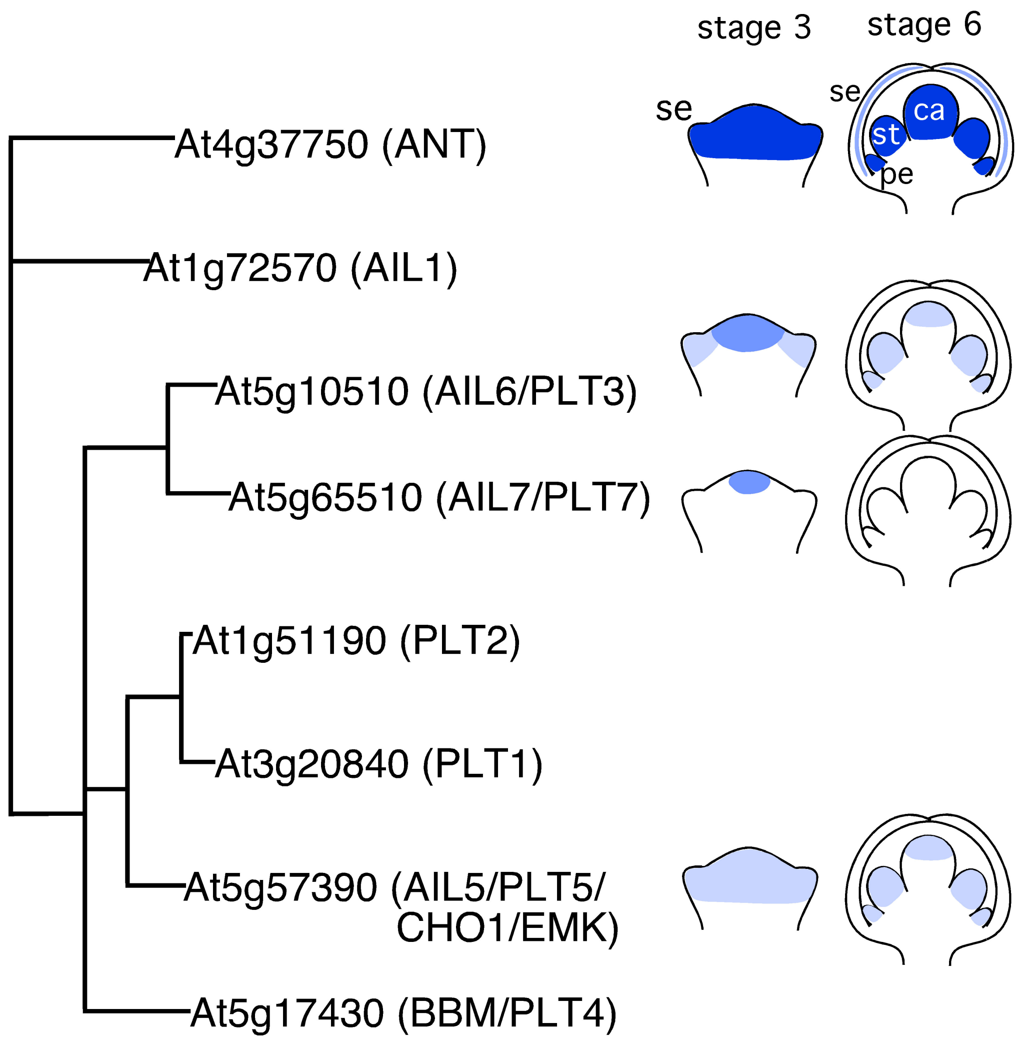

Supplementary Figure S1. AIL family tree and summary of gene expression data. Phylogenetic tree showing the relationship among the eight AIL proteins (left). Some AIL proteins have multiple names. AIL6 is also known as PLETHORA6 (PLT6) (Galinha *et al.*, 2007). AIL7 is also known as PLETHORA7 (PLT7) (Prasad *et al.*, 2011). AIL5 is also known as PLETHORA5 (PLT5), CHOTTO1 (CHO1) and EMBRYOMAKER (EMK) (Prasad *et al.*, 2011; Tsuwamoto *et al.*, 2010; Yamagishi *et al.*, 2009). BABYBOOM (BBM) is also known as PLETHORA4 (PLT4) (Prasad *et al.*, 2011). Summary of mRNA expression patterns for *ANT*, *AIL5*, *AIL6* and *AIL7* in stage 3 and stage 6 flowers (right) as determined by in situ hybridization (Elliott *et al.*, 1996; Nole-Wilson *et al.*, 2005). Sepal primordia are initiated in stage 3 flowers while sepal, petal, stamen and carpel primordia are present in stage 6 flowers. The intensity of the blue color represents gene expression levels with a darker color indicating higher expression.

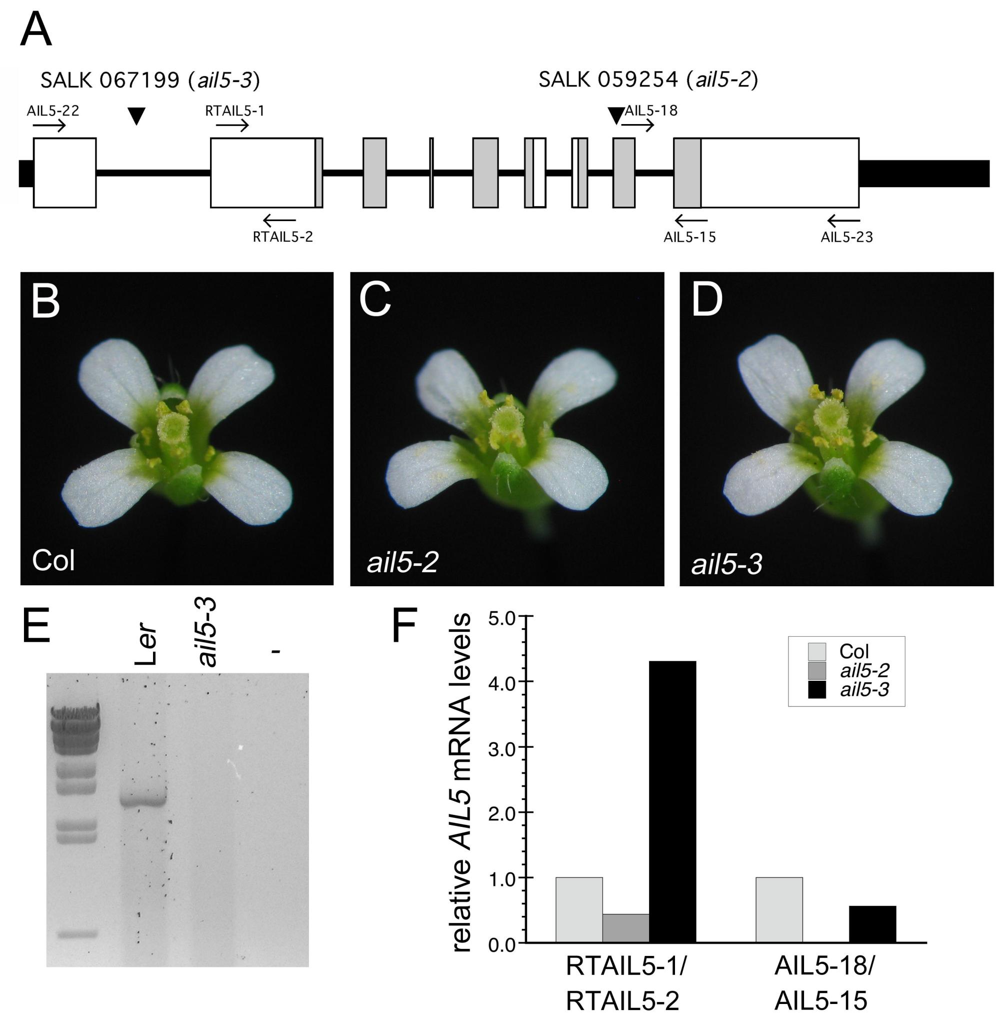

Supplementary Figure S2. *ail5* mutants have a wild-type appearance. A. *AIL5* gene structure with exons indicated by boxes (white and grey), introns indicated by thin lines and the 5' and 3' UTRs indicated with black boxes. The two AP2 domains are shown in grey. The positions of the T-DNA insertions in SALK 067199 (*ail5-3*) and SALK 059254 (*ail5-2*) are indicated with triangles. Primers used for RT-PCR (AIL5-22/AIL5-23) and real time PCR (RTAIL5-1/RTAIL5-2 and AIL5-18/AIL5-15) are indicated by arrows above and below the gene. B. Col flower. C. *ail5-2* flower. D. *ail5-3* flower. E. RT-PCR gel showing that full-length *AIL5* transcript (1.7kb) is present in L*er* but not *ail5-3 er*. F. Graph of real time PCR results showing that transcripts corresponding to regions downstream of the T-DNA were detected in *ail5-3 ER* plants while transcripts corresponding to regions upstream of the T-DNA (but not downstream) were detected in *ail5-2* ER plants.

Supplementary Table S1. Primers used in this study

Name	Primer sequence (5' to 3')	Purpose
AIL5-14	TGAAATGATTACCCAACCGTG	genotype *ail5-2*
AIL5-15	TAGGCATTAGTCCACCCACAG	genotype *ail5-2*
AIL5-16	TCGAGGTACGTTGTCCAAAAC	genotype *ail5-3*
AIL5-17	TTCCAAAGTCGTGAAATCACC	genotype *ail5-3*
AIL6-36	CTGTTTTGTGATTTGCCATTG	genotype *ail6-2*
AIL6-37	AAGCCTAGATCCCCACTCTTG	genotype *ail6-2*
AIL7-14	GTCATACCTGTTCATCTCAAAGT	genotype *ail7-1*
AIL7-36	GCCTTGACCCTAATCAAATCC	genotype *ail7-1*
LBb1.3	ATTTTGCCGATTTCGGAAC	genotype *ail5-2, ail5-3*
LB1	GCCTTTTCAGAAATGGATAAATAGCCTTGCTTCC	genotype *ail6-2, ail7-1*
AIL5-22	ATGAAGAACAATAACAACAAATCT	RT-PCR *ail5-3*
AIL5-23	TCATTCCAACCCAAAAACCG	RT-PCR *ail5-3*
RTAIL5-1	CTACTCCGGTGGACACTCGT	real time PCR *ail5-2* and *ail5-3*
RTAIL5-2	CGTTCTTCTTCGGAGTAGGC	real time PCR *ail5-2* and *ail5-3*
AIL5-18	CAGGCACGAATTGGAAGAGTTG	real time PCR *ail5-2* and *ail5-3*
AIL5-15	TAGGCATTAGTCCACCCACAG	real time PCR *ail5-2* and *ail5-3*

References

Elliott RC, Betzner AS, Huttner E, Oakes MP, Tucker WQJ, Gerentes D, Perez P, Smyth DR. 1996. *AINTEGUMENTA*, an *APETALA2*-like gene of *Arabidopsis* with pleiotropic roles in ovule development and floral organ growth. *The Plant Cell* **8**, 155-168.

Galinha C, Hofhuis H, Luijten M, Willemsen V, Blilou I, Heidstra R, Scheres B. 2007. PLETHORA proteins as dose-dependent master regulators of Arabidopsis root development. *Nature* **449**, 1053-1057.

Nole-Wilson S, Tranby T, Krizek BA. 2005. *AINTEGUMENTA*-like (*AIL*) genes are expressed in young tissues and may specify meristematic or division-competent states. *Plant Molecular Biology* **57**, 613-628.

Prasad K, Grigg SP, Barkoulas M, Yadav RK, Sanchez-Perez GF, Pinon V, Blilou I, Hofhuis H, Dhonukshe P, Galinha C, Mahonen AP, Muller WH, Raman S, Verkleij AJ, Snel B, Reddy GV, Tsiantis M, Scheres B. 2011. Arabidopsis PLETHORA transcription factors control phyllotaxis. *Current Biology* **21**, 1123-1128.

Tsuwamoto R, Yokoi S, Takahata Y. 2010. *Arabidopsis EMBRYOMAKER* encoding an AP2 domain transcription factor plays a key role in developmental change from vegetative to embryonic phase. *Plant Molecular Biology* **73**, 481-492.

Yamagishi K, Tatematsu K, Yano R, Preston J, Kitamura S, Takahashi H, McCourt P, Kamiya Y, Nambara E. 2009. CHOTTO1, a double AP2 domain protein of Arabidopsis thaliana, regulates germination and seedling growth under excess supply of glucose and nitrate. *Plant and Cell Physiology* **50**, 330-340.
